# A multicenter RCT of noninvasive ventilation in pneumonia-induced early mild acute respiratory distress syndrome

**DOI:** 10.1186/s13054-019-2575-6

**Published:** 2019-09-04

**Authors:** Hangyong He, Bing Sun, Lirong Liang, Yanming Li, He Wang, Luqing Wei, Guofeng Li, Shuliang Guo, Jun Duan, Yuping Li, Ying Zhou, Yusheng Chen, Hongru Li, Jingping Yang, Xiyuan Xu, Liqiang Song, Jie Chen, Yong Bao, Feng Chen, Ping Wang, Lixi Ji, Yongxiang Zhang, Yanyan Ding, Liangan Chen, Ying Wang, Lan Yang, Tian Yang, Heng Weng, Hongyan Li, Daoxin Wang, Jin Tong, Yongchang Sun, Ran Li, Faguang Jin, Chunmei Li, Bei He, Lina Sun, Changzheng Wang, Mingdong Hu, Xiaohong Yang, Qin Luo, Jin Zhang, Hai Tan, Chen Wang, for the ENIVA Study Group, Hangyong He, Bing Sun, Lirong Liang, Yanming Li, He Wang, Luqing Wei, Guofeng Li, Bin Liu, Shuliang Guo, Jun Duan, Yuping Li, Ying Zhou, Yusheng Chen, Hongru Li, Rujun Hong, Xiujuan Yao, Fengfeng Lu, Jingping Yang, Xiyuan Xu, Hui Wang, Ling Wang, Hongjun Tian, Liqiang Song, Jie Chen, Yunfu Wu, Yong Bao, Feng Chen, Ping Wang, Lixi Ji, Xiaofang Huang, Min Sun, Yongxiang Zhang, Yanyan Ding, Liangan Chen, Ying Wang, Zhixin Liang, Lan Yang, Tian Yang, Heng Weng, Hongyan Li, Xu Lin, Daoxin Wang, Jin Tong, Wang Deng, Yongchang Sun, Ran Li, Jie Xu, Faguang Jin, Yandong Nan, Chunmei Li, Bei He, Ning Shen, Lina Sun, Changzheng Wang, Mingdong Hu, Xiaohong Yang, Qin Luo, Mei Li, Jin Zhang, Hai Tan, Chen Wang

**Affiliations:** 1 Department of Respiratory and Critical Care Medicine, Beijing Institute of Respiratory Medicine, Beijing Key Laboratory of Respiratory and Pulmonary Circulation Disorders, Beijing Engineering Research Center for Diagnosis and Treatment of Pulmonary and Critical Care, Beijing Chao-Yang Hospital, Capital Medical University, No. 8 Gongren Tiyuchang Nanlu, Chaoyang District, Beijing, 100020 China; 2 Department of Respiratory and Critical Care Medicine, Beijing Hospital, Beijing, China; 3 Department of Respiratory and Critical Care Medicine, Affiliated Hospital of Logistics College of Chinese Armed Police Forces, Tianjin, China; 4 Department of Respiratory and Critical Care Medicine, The First Affiliated Hospital of Chongqing Medical University, Chongqing, China; 5 Department of Respiratory and Critical Care Medicine, The First Affiliated Hospital of Wenzhou Medical University, Wenzhou, Zhejiang Province China; 6 The Pulmonary Department, Fujian Province Hospital, Fuzhou, Fujian Province China; 7 Department of Respiratory and Critical Care Medicine, The Third Affiliated Hospital of Inner Mongolia Medical College, Baotou, Inner Mongolia Autonomous Region China; 8 The Pulmonary Department, Xijing Hospital of the Fourth Military Medical University, Xi’an, Shanxi Province China; 9 The Pulmonary Department, The Third People’s Hospital of Chengdu, Chengdu, Sichuan Province China; 10 Department of Critical Care Medicine, Chengdu Fifth People’s Hospital, Chengdu, Sichuan Province China; 11 Department of Respiratory Medicine, People’s Hospital of Beijing Daxing District, Beijing, China; 12 Department of Pulmonary and Critical Care Medicine, Chinese PLA General Hospital, Beijing, China; 13 Department of Respiratory and Critical Care Medicine, The First Affiliated Hospital of Xi’an Jiaotong University, Xi’an, Shanxi Province China; 14 The Pulmonary Department, Lung Disease Hospital of Fujian Fuzhou, Fuzhou, Fujian Province China; 15 The Pulmonary Department, The Second Affiliated Hospital of Chongqing Medical University, Chongqing, China; 16 The Pulmonary Department, Beijing Tongren Hospital, Beijing, China; 17 Department of Respiratory and Critical Care Medicine, Tangdu Hospital, the Fourth Military Medical University, Xi’an, Shanxi Province China; 18 The Pulmonary Department, Peking University Third Hospital, Beijing, China; 19 The Pulmonary Department, Xinqiao Hospital Army Medical University, Chongqing, China; 20 Department of Respiratory and Critical Care Medicine, People’s Hospital of Xinjiang Uygur Autonomous Region, Urumqi, Xinjiang Uygur Autonomous Region China; 21 Department of Respiratory and Critical Care Medicine, General Hospital of Ningxia Medical University, Yinchuan, Ningxia Province China; 22 Chinese Academy of Medical Sciences and Peking Union Medical College, Beijing, China; 23 Department of Pulmonary and Critical Care Medicine, Center of Respiratory Medicine, China-Japan Friendship Hospital, No.2 Yinghua East Road, Chaoyang District, Beijing, 100029 China; 24 Department of Respiratory Medicine, Capital Medical University, Beijing, China; 25 National Clinical Research Center for Respiratory Diseases, Beijing, China

**Keywords:** Acute respiratory distress syndrome (ARDS), Noninvasive ventilation (NIV), Pneumonia

## Abstract

**Rationale:**

Our pilot study suggested that noninvasive ventilation (NIV) reduced the need for intubation compared with conventional administration of oxygen on patients with “early” stage of mild acute respiratory distress syndrome (ARDS, PaO_2_/FIO_2_ between 200 and 300).

**Objectives:**

To evaluate whether early NIV can reduce the need for invasive ventilation in patients with pneumonia-induced early mild ARDS.

**Methods:**

Prospective, multicenter, randomized controlled trial (RCT) of NIV compared with conventional administration of oxygen through a Venturi mask. Primary outcome included the numbers of patients who met the intubation criteria.

**Results:**

Two hundred subjects were randomized to NIV (*n* = 102) or control (*n* = 98) groups from 21 centers. Baseline characteristics were similar in the two groups. In the NIV group, PaO_2_/FIO_2_ became significantly higher than in the control group at 2 h after randomization and remained stable for the first 72 h. NIV did not decrease the proportion of patients requiring intubation than in the control group (11/102 vs. 9/98, 10.8% vs. 9.2%, *p* = 0.706). The ICU mortality was similar in the two groups (7/102 vs. 7/98, 4.9% vs. 3.1%, *p* = 0.721). Multivariate analysis showed minute ventilation greater than 11 L/min at 48 h was the independent risk factor for NIV failure (OR, 1.176 [95% CI, 1.005–1.379], *p* = 0.043).

**Conclusions:**

Treatment with NIV did not reduce the need for intubation among patients with pneumonia-induced early mild ARDS, despite the improved PaO_2_/FIO_2_ observed with NIV compared with standard oxygen therapy. High minute ventilation may predict NIV failure.

**Trial registration:**

NCT01581229. Registered 19 April 2012

**Electronic supplementary material:**

The online version of this article (10.1186/s13054-019-2575-6) contains supplementary material, which is available to authorized users.

## Introduction

Acute respiratory distress syndrome (ARDS) mortality ranges from 35 to 46%. Mortality is related to the severity of ARDS and remains high despite improvement in recent years [1]. Noninvasive positive-pressure ventilation (hereafter, noninvasive ventilation, NIV) reduces the need for endotracheal intubation and mortality among patients with acute respiratory failure [2, 3], but its use in ARDS is uncertain [4].

Previous studies often included a heterogeneous population of patients with ARDS caused by pulmonary infection, sepsis, acute pancreatitis, or multiple trauma; this selection of patients could lead to an overestimation of the beneficial effects of NIV as compared with standard oxygen therapy. Pneumonia is a major cause of pulmonary ARDS. In observational ARDS studies, the rate of treatment failure with NIV was as high as 50% [5–7] and associated with particularly high mortality in pulmonary infection-induced ARDS [8]. Currently, NIV use in ARDS remains highly controversial [9–11], especially in pneumonia-induced ARDS.

Although more than half of mild ARDS cases (arterial oxygen tension/inspired oxygen fraction [PaO_2_/FIO_2_] ≤ 300 mmHg but > 200 mmHg) rapidly evolve to moderate or severe ARDS [12], many of these patients may not require invasive mechanical ventilation with the lower severity of mild ARDS. Our pilot study [13] suggests that NIV for patients with mild ARDS reduced the need for intubation and the number of organ failures compared with conventional administration of oxygen through a Venturi mask. In this study, NIV also reduced the need for intubation in pneumonia-induced mild ARDS (10% vs. 50%). However, because of the small sample size and the etiological heterogeneity of this study, the benefit of NIV versus oxygen in pneumonia-induced early mild ARDS needs confirmation in a trial with a large sample size and homogeneous population [14].

We hypothesized that more severe hypoxemia and comorbidities are the primary causes of NIV failures in pulmonary infection-induced ARDS. We therefore designed a multicenter randomized controlled trial to test the hypothesis that initiating NIV during early mild ARDS induced by pneumonia could prevent patients from evolving to moderate/severe ARDS and decrease the need for invasive mechanical ventilation compared with oxygen only.

## Methods

### Patient selection

All patients admitted to a hospital ward or ICU with pneumonia were screened. Eligible subjects were ≥ 18 years of age with pneumonia-induced early mild ARDS. Key inclusion criteria were clinical diagnosis of pneumonia, bilateral radiographic infiltrates on chest radiograph, acute onset with worsen respiratory status, and mild hypoxemia defined as 200 mmHg < PaO_2_/FIO_2_ < 300 mmHg while breathing oxygen delivered by a conventional Venturi device at a fraction of inspiration oxygen of 0.5 [13, 15]. Patients with contraindications of NIV, severe organ failure, unable to cooperate with NIV, or ARDS caused by extra-pulmonary reasons were excluded (complete criteria for pneumonia, inclusion and exclusion provided in Additional file 1: 1.1 Section S1).

### Study design

This prospective randomized, controlled trial (NCT01581229) enrolled patients at 21 departments of respiratory and critical care medicine of 21 university-affiliated hospitals across 10 provinces in Mainland China. All of these departments are members of a collaborating study group for NIV in China (ENIVA Study Group) and have experience with multicenter clinical trials for NIV [13]. The study was approved by the ethics committees of all participating institutions. All participating subjects or their next of kin provided written informed consent. Within 24 h of fulfilling inclusion criteria, a patient was randomly allocated either to the NIV group or the control group (Venturi mask oxygen therapy). This definition of early mild ARDS was used to avoid the issue of returning to oxygen therapy alone after randomization if every patient was required to receive PEEP or CPAP greater than 5 cmH_2_O to meet the Berlin ARDS definition prior to randomization (details for randomization and quality control were provided in Additional file 1: 1.2 and 1.3 Section S2 and S3).

#### Noninvasive ventilation

Patients in the NIV group were ventilated using the bilevel positive airway pressure S/T mode (BiPAP Vision or V60; Respironics Inc., Murrysville, PA). The same oral-nasal face mask (ZS-MZ-A Face Mask; Shanghai Zhongshan Medical Technology Co., Shanghai, China) was used for all patients. NIV was delivered for no less than 16 h a day in the first 3 days after entry into the study. Setting and adjusting of expiratory positive airway pressure (EPAP), FIO_2_, and inspiratory positive airway pressure (IPAP) and disconnecting and withdraw from NIV followed the previous study protocol [13](NIV protocol was provided in Additional file 1: 1.4 Section S4).

#### Conventional oxygen therapy

In the control group, Venturi masks were used to maintain SpO_2_ at 92 to 96% by adjusting the oxygen flow rates and FIO_2_.

#### Endotracheal intubation

Intubation was considered if a patient failed to maintain a PaO_2_/FIO_2_ of > 200 mmHg despite optimal standard oxygen therapy or NIV and at least two of the following criteria were met: (1) respiratory rate ≥ 35 breaths/min; (2) blood pH < 7.30; (3) a score of 3–5 on the Kelly scale of neurologic dysfunction; and (4) a score of ≥ 3 points on a modified scale of accessory respiratory muscle use(see criteria in Additional file 1: 1.4 Section S4) [13]. Once a patient fulfilled these criteria, the final decision for intubation was made by the attending physician with consent of the family members, which meant that the patients in both groups who met the intubation criteria were considered failed and could be intubated or crossed over to NIV in the control group.

Comprehensive therapy: The treatment of pneumonia is followed by the protocol provided in Additional file 1: 1.4 Section S4. Comprehensive therapy was provided by the ICU attending physicians based on published guidelines.

### End points and measurements

The primary end point was the number of patients who met the intubation criteria.

The secondary end points included ICU and in-hospital mortality; complications resulting from invasive mechanical ventilation, including barotrauma and ventilator-associated pneumonia; rates of hospital-acquired infections and organ failures; and lengths of ICU and hospital stays.

Other variables collected included (1) Acute Physiology and Chronic Health Evaluation II (APACHE II) scores on study entry; (2) respiratory rate, heart rate, mean arterial pressure, SpO_2_, and arterial blood gas analysis results on study entry and after 2, 12, 24, 48, and 72 h; (3) routine blood and blood biochemistry results on study entry and after 24, 48, and 72 h; and (4) NIV parameters, including IPAP and EPAP levels, hours of NIV use each day, total NIV duration, and NIV complications.

### Statistical analysis

#### Sample size estimation

Based on the intubation rate for control patients (36.8%) and pneumonia-induced mild ARDS (50%) reported in our previous pilot study [13], we estimated that a total of 184 subjects (92 in each group) were required with an expected intubation rate of 40% in the control group and of 32% (40% × (1–0.2) = 32%, a 20% reduction) in the NIV group (confidence level [1 − *α*] = 95% and power level[1 − *β*] = 80%), and with a maximum drop-out rate of 15%.

#### Comparisons between the two groups

Quantitative continuous variables were given as either means (± SDs) or medians (with inter-quartile ranges) that were compared using the unpaired Student’s *t* test or the Mann-Whitney test. Qualitative or categorical variables were compared with the chi-square test or Fisher’s exact test. ANOVA for paired tests to compare the same variables collected at different time points was used. The cumulative probability of remaining on spontaneous breathing was compared with the Kaplan-Meier estimate of survival and the log-rank test to compare the two groups. Univariate and multivariate analyses of risk factors for NIV failure were performed with logistic regression. All analyses were in intention to treat, and the level of significance was set at 0.05.

## Results

### Patients’ characteristics

**Fig. 1:**
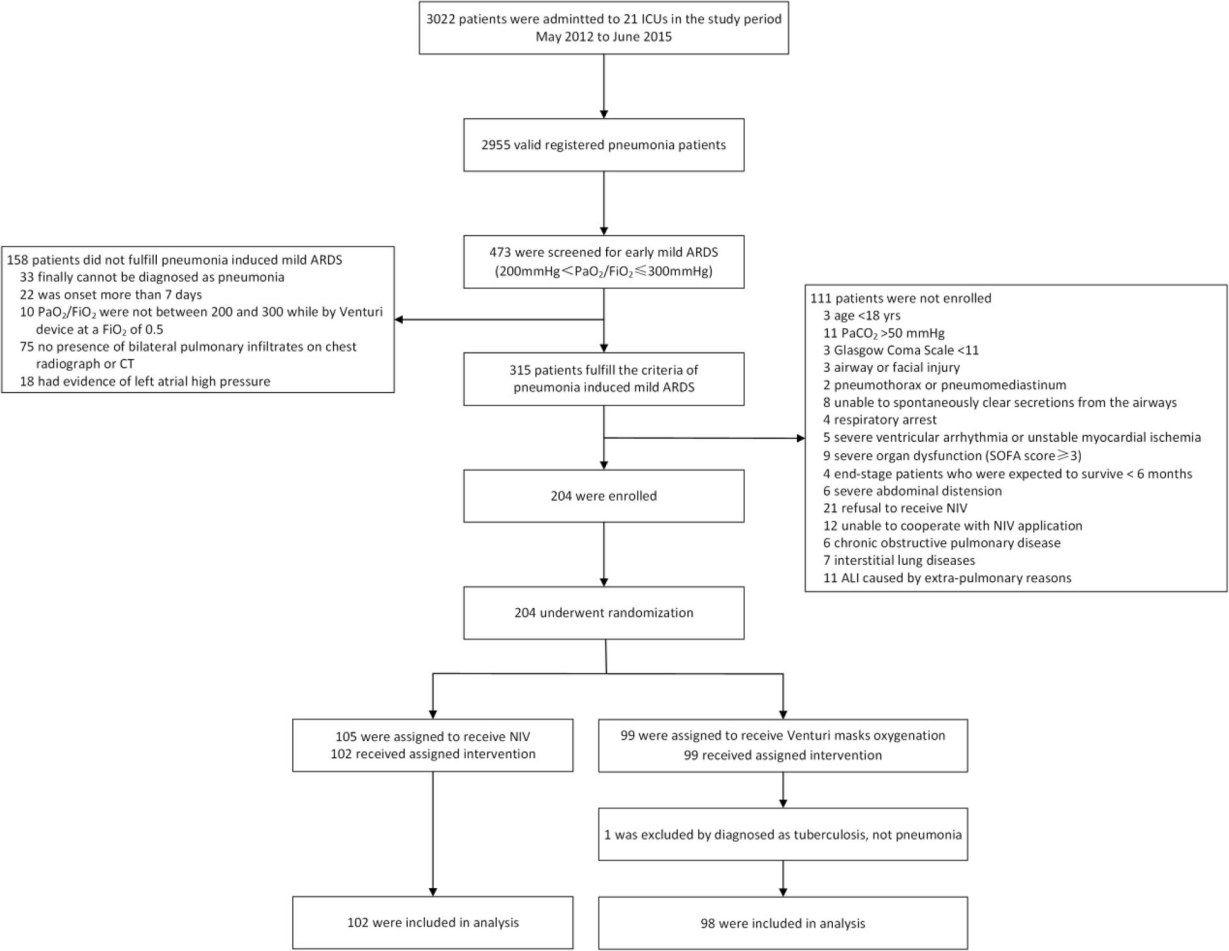
Flow diagram for the trial. ICU, intensive care unit; NIV, noninvasive ventilation; ALI, acute lung injury; PaO_2_/FIO_2_, arterial oxygen tension/inspired oxygen fraction; CT, computed tomography; SOFA, sequential organ failure assessment; PaCO_2_, arterial pressure of carbon dioxide

Between May 2012 and June 2015, 3022 pneumonia patients were admitted to the 21 centers of which 2955 patients had valid data; of these, 473 patient’s PaO_2_/FIO_2_ ratios were between 200 and 300, 315 patients fulfill the criteria of early mild ARDS, 111 patients had exclusion criteria, thus 204 patients were enrolled. One hundred five were allocated to the NIV group and 99 to the control group. Three patients refused NIV after randomization to the NIV group, and 1 patient was diagnosed as having tuberculosis in the control group. Therefore, 200 patients were included in the final analysis (Fig. 1).

Baseline characteristics of the two groups were similar (Table 1). Among underlying diseases, only the incidence of diabetes mellitus differed between the two groups (*p* = .014). Type of pneumonia was community-acquired for 91.5% in the NIV group and 94.9% in the control group. Lower respiratory tract sample cultures were positive for bacteria in 9.4% and 16% patients and were positive for fungus in 16.7% and 8.5% patients in the NIV and control groups, respectively. Empirical antibiotics were used at inclusion for suspected bacteria, *Legionella*, mycoplasma/Chlamydia, virus, and fungus (including pneumocystis) in 90.5%, 10%, 15%, 29%, and 21.5% (11%) patients, respectively.

**Table 1:** Patient baseline characteristics

	NIV group (*n* = 102)	Control group (*n* = 98)	*p* value
Age, years, mean (SD)	53 (18.2)	56 (17.5)	0.21
Male, no. (%)	69 (66.8)	62 (64.2)	0.52
Smoking, no. (%)	34 (33.3)	25 (25.5)	0.23
Body mass index, kg/m^2^, mean (SD)	22.4 (3.2)	22.3 (4.5)	0.93
Type of pneumonia, no. (%)			
Community-acquired	95 (93.1)	93 (94.9)	0.60
Hospital-acquired	7 (6.9)	5 (5.1)	0.60
Positive culture of pathogen, no. (%)	23 (22.5)	7 (7.1)	0.01
Bacteria from the respiratory sample	9 (9.4)	15 (16.0)	0.17
Blood culture	2 (2.0)	4 (4.2)	0.37
Fungus from the respiratory sample	13 (16.7)	6 (8.5)	0.13
Underlying comorbidities, no. (%)			
Hypertension	25 (24.5)	23 (23.5)	0.86
Diabetes mellitus	7 (6.9)	18 (18.4)	0.01
Coronary heart disease	6 (5.9)	5 (5.1)	0.81
Chronic heart failure	1 (1.0)	2 (2.0)	0.54
Chronic renal insufficiency	4 (3.9)	8 (8.2)	0.21
Cancer	4 (3.9)	2 (2.0)	0.44
Cerebrovascular disease	2 (2.0)	7 (7.1)	0.08
Immunosuppression	9 (8.8)	10 (10.2)	0.74
White blood cell count, × 10^9^/L, mean (SD)	10.7 (5.6)	10.2 (5.9)	0.56
Neutrophil, × 10^9^/L, mean (SD)	80.7 (14.6)	82.7 (12.6)	0.29
Arterial blood gas analysis			
pH, mean (SD)	7.445 (0.052)	7.453 (0.051)	0.24
PaO_2_/FiO_2_, mmHg, mean (SD)	231.5 (35.0)	231.3 (27.8)	0.96
PaCO_2_, mmHg, mean (SD)	34.5 (5.7)	33.5 (4.8)	0.19
Biochemistry examination			
Albumin, g/L, mean (SD)	31.3 (5.9)	30.5 (6.6)	0.37
Alanine aminotransferase, U/L, mean (SD)	53.7 (62.3)	47.1 (49.7)	0.41
Aspartate aminotransferase U/L, mean (SD)	61.7 (107.8)	57.4 (75.1)	0.74
nT-pro BNP, pg/mL, mean (SD)	1660 (5476)	1270 (2363)	0.64
C-reactive protein, mg/dL, mean (SD)	84.3 (84.8)	99.3 (160.5)	0.41
Procalcitonin, ng/mL, mean (SD)	46.5 (211.8)	12.6 (47.0)	0.12
APACHE II score, mean (SD)	7.0 (4.3)	8.1 (4.2)	0.14
Admitted to ICU, no. (%)	73(71.6)	79 (80.6)	0.13

*NIV* noninvasive ventilation, *ICU* intensive care unit, *APACHE II score* Acute Physiology and Chronic Health Evaluation II score, *PaO*_*2*_ partial pressure of arterial oxygen, *FiO*_*2*_ fraction of inspired oxygen

Eighty-four cases (82.4%, *n* = 84/102) had a PaO2/FiO2 ratio lower than 300 in the NIV group patients at 1 h after receiving NIV. This means that most of the patients we included as an early mild ARDS also fulfilled the Berlin definition of mild ARDS after a positive pressure was used.

### NIV application

The average period of NIV was 6.3 ± 3.7 days (range, 1–20 days). The average daily treatment time for NIV at the first 3 days was day 1 = 17.3 h (range 1–24, *n* = 102), day 2 = 18.2 h (range 4–24, *n* = 97), and day 3 = 16.8 h (range 4–24, *n* = 94). The number of patients receiving NIV and daily ventilation time is shown in Additional file 1: 3.1 Figure S1. Levels of IPAP (actual inspiratory pressure, not pressure above EPAP) and EPAP were 14 (range, 9–26) cmH_2_O and 6 (range, 4–11) cmH_2_O, respectively (in Additional file 1: 3.2 Figure S2).

Complications associated with NIV were observed in 16 patients (15.6%), with 9 cases of cough suppression and inefficient cough as the most frequent complication (8.8%). One patient had NIV discontinued because of cough suppression and subsequently refused to receive further NIV. Five cases each of abdominal distension and facial abrasion, 4 cases of severe air-leak, and 1 case of aspiration occurred. No barotrauma was reported. Eleven patients ceased NIV when they met intubation criteria; in these patients, 4 patients had NIV complications (2 abdominal distension, 1 facial abrasion, and 1 with abdominal distension, facial abrasion, aspiration, and cough suppression).

### Primary end point

The number of patients who met intubation criteria was 11 and 9 in the NIV group and the control group, respectively. Two patients in each group refused to intubation. No significant differences were found between the two treatment groups in the need for intubation rate (10.8% vs. 9.2%, *p* = 0.71) or actual intubation rate (8.8% vs. 7.1%, *p* = 0.66). The two patients who refused intubation in the control group received NIV rescue therapy (Table 2).

**Table 2:** Main outcomes

	NIV group (*n* = 102)	Control group (*n* = 98)	*p* value
Need for intubation, no. (%)	11 (10.8)	9 (9.2)	0.71
Intubation, no. (%)	9 (8.8)	7 (7.1)	0.66
Duration to intubation, days, mean (SD)	4.7 (6.7)	2.6 (2.9)	0.38
Reason for intubation			
PaO_2_/FiO_2_ at intubation, mmHg, mean (SD)	120.0 (41.9)	147.6 (35.7)	0.13
RR at intubation, bpm, mean (SD)	43.8 (7.2)	39.4 (3.6)	0.13
Patients RR > 35 bpm at intubation, no. (%)	10 (90.9)	8 (88.9)	1.00
Patients’ pH < 7.35 at intubation, no. (%)	4 (36.4)	1 (11.1)	0.32
Kelly score > 3 at intubation, no. (%)	4 (36.4)	3 (33.3)	1.00
Accessory respiratory muscle, no. (%)	8 (72.7)	6 (66.7)	1.00
Mortality			
Death in hospital, no. (%)	7 (6.9)	7 (7.1)	0.95
Death in ICU, no. (%)	7 (6.9)	7 (7.1)	0.72
Death of intubated patients, no. (%)	7 (63.6)	7 (77.8)	0.64
Days of intensive care, days, mean (SD)	11.4 (7.5)	8.6 (5.3)	0.22
Days of hospital, days, mean (SD)	16.5 (9.4)	17.2 (10.3)	0.65
Cost for hospitalization, RMB, mean (SD)	47,273 (40965)	60,115 (68418)	0.11
Complications			
Hospital-acquired infection, no. (%)	7 (6.9)	4 (4.1)	0.39
Hospital-acquired pneumonia	4 (3.9)	2 (2.0)	0.68
Catheter-related infection	2 (2.0)	1 (1.0)	1.00
Organ failure, no. (%)	8 (7.8)	13 (13.3)	0.21
Renal failure	3 (2.9)	4 (4.1)	0.72
Cardiovascular failure	4 (3.9)	8 (8.2)	0.21
Hepatic failure	4 (3.9)	4 (4.1)	1.00
Hematologic failure	5 (4.9)	4 (4.1)	1.00
Central nervous system failure	1 (1.0)	2 (2.0)	0.62

*NIV* noninvasive ventilation, *RR* respiratory rate, *PaO*_*2*_ partial pressure of arterial oxygen, *FiO*_*2*_ fraction of inspired oxygen

The average duration between inclusion and intubation were 4.7 days and 2.6 days for NIV and control groups, respectively. The indications for endotracheal intubation were similar in the two groups. The main reason for intubation was severe hypoxemia, high RR, and fatigue of the respiratory muscle.

Subgroup analysis for white blood cell count, neutrophil cell percentage, respiratory rate, immunocompromised state, procalcitonin level, and types of pneumonia at inclusion found no difference between NIV and control groups for intubation rate (see in Additional file 1: 2.1 Table S1).

### Secondary end points

**Fig. 2:**
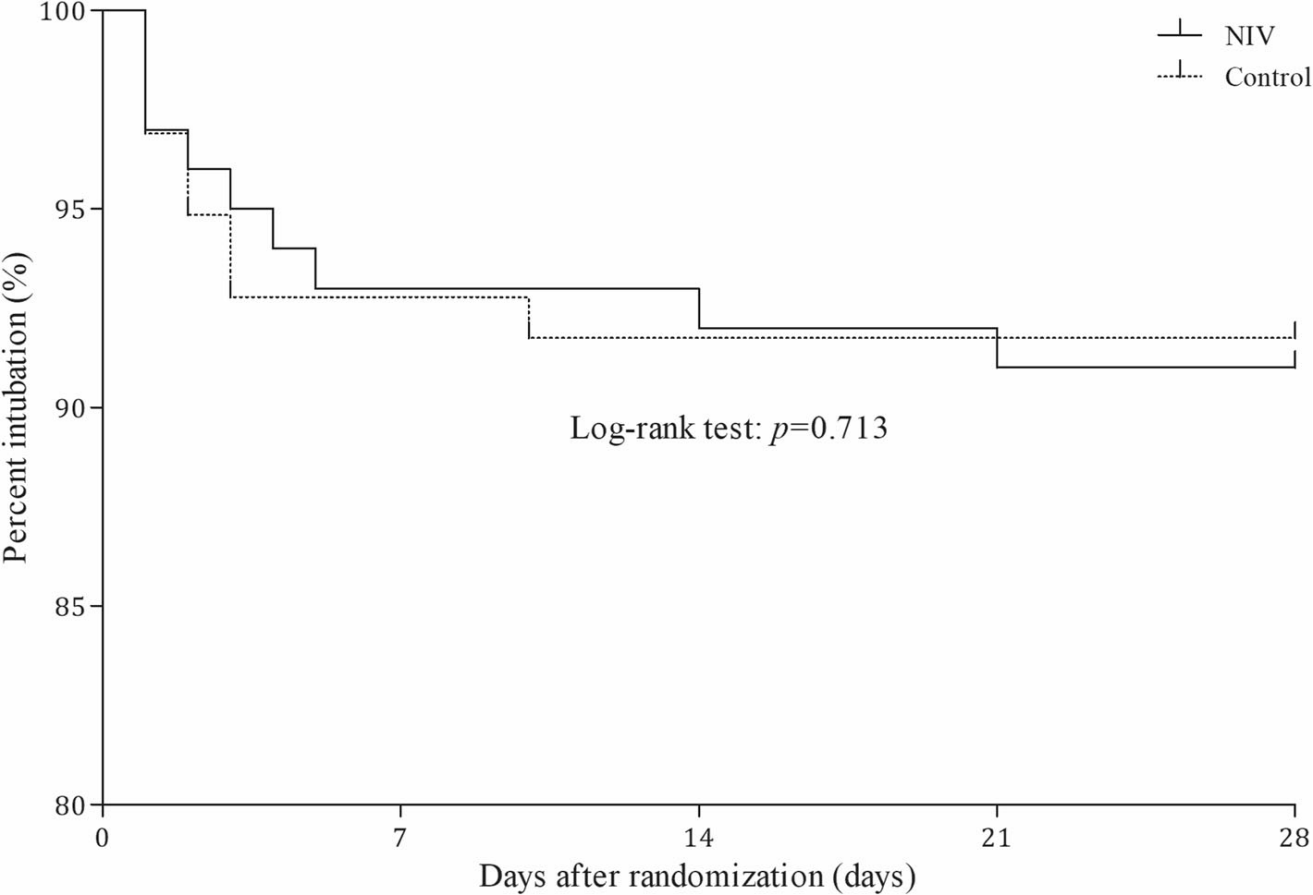
Kaplan-Meier estimates of the probability of the need for endotracheal intubation based on the criteria for endotracheal intubation. No difference was found for the cumulative probability for endotracheal intubation of the two groups (log-rank test: *p* = 0.71)

**Fig. 3:**
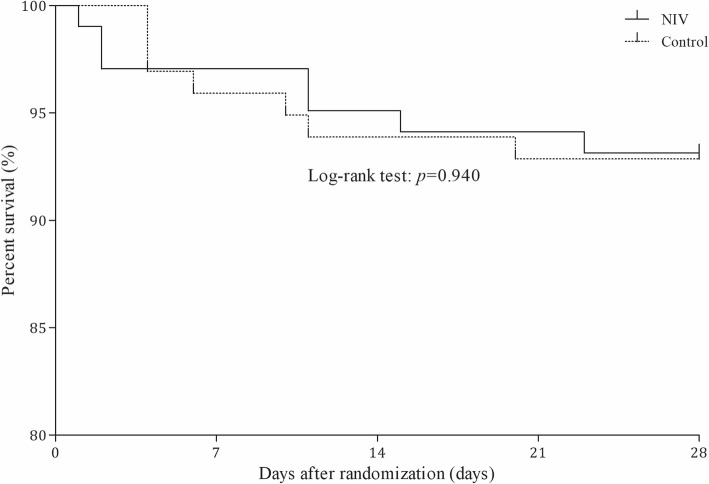
Kaplan-Meier estimates of the probability of mortality. No difference was found for the cumulative probability for endotracheal intubation of the two groups (log-rank test: *p* = 0.94)

The ICU mortality and in-hospital mortality were similar in the NIV group and the control group (Table 2). No statistically significant difference in need for intubation and overall in-hospital mortality was found between the two groups by log-rank test (Figs. 2 and 3). No significant differences were found between the two treatment groups for any of the other secondary end point variables, including blood pressure, heart rate, complications, number of organ failures, costs in hospital, and lengths of ICU and hospital stays (Table 2).

### Time course of PaO_2_/FiO_2_ ratio, arterial blood gases, and respiratory frequency

**Fig. 4:**
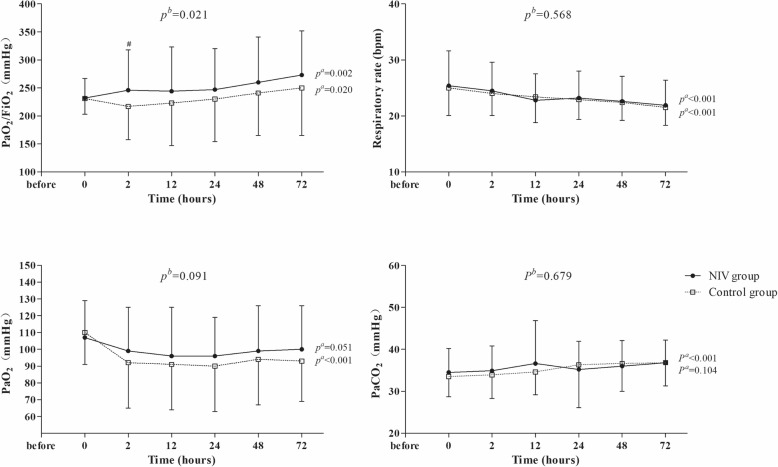
Comparisons of physiological parameters between noninvasive ventilation and control groups

As shown in Table 3 and Fig. 4, an improvement of PaO_2_/FIO_2_overtime occurred in both groups. In the NIV group, PaO_2_/FIO_2_ became significantly higher than in the control group at 2 h after randomization and remained stable for the first 72 h. The FiO_2_ in the two groups shows a similar value and trend as 0.5 in the baseline and 0.4 in the first 24 h and 0.35 to 0.4 within 24 to 72 h. The respiratory rates improved with time in both groups. No differences between the two groups existed for the time course of arterial pH, PaCO_2_, or PaO_2_.

**Table 3:** Comparisons of physiological parameters between noninvasive ventilation and control groups

Variables	Group	Baseline (*n* = 102/98)	2 h (*n* = 102/98)	12 h (*n* = 102/98)	24 h (*n* = 99/95)	48 h (*n* = 98/94)	72 h (*n* = 96/93)	*p* ^a^
PaO_2_/FiO_2_, mmHg	NIV	232 (35)	246 (72)	244 (79)	247 (73)	260 (81)	273 (79)	.00
	Control	231 (28)	217 (59)	223 (76)	230 (76)	241 (76)	250 (85)	.02
	*p* ^c^	.96	.00	.06	.12	.09	.06	.02^b^
pH	NIV	7.45 (0.05)	7.44 (0.05)	7.44 (0.04)	7.37 (0.76)	7.44 (0.05)	7.44 (0.04)	.82
	Control	7.45 (0.05)	7.45 (0.05)	7.44 (0.05)	7.44 (0.05)	7.44 (0.04)	7.44 (0.04)	.56
	*p* ^c^	.24	.42	.99	.34	.33	.65	.31^b^
PaCO_2_, mmHg	NIV	34.5 (5.7)	34.9 (5.9)	36.6 (10.2)	35.2 (6.7)	36.0 (6.1)	36.8 (5.4)	.11
	Control	33.5 (4.8)	33.9 (5.6)	34.6 (5.4)	36.3 (10.2)	36.6 (6.6)	36.8 (5.5)	.00
	*p* ^c^	.19	.26	.09	.39	.52	.98	.68^b^
PaO_2_, mmHg	NIV	107 (22)	99 (26)	96 (29)	96 (23)	99 (27)	100 (26)	.05
	Control	110 (19)	92 (27)	91 (27)	90 (27)	94 (27)	93 (24)	.00
	*p* ^c^	.17	.06	.19	.13	.24	.07	.09^b^
RR, breaths/min	NIV	25.4 (6.2)	24.5 (5.1)	22.8 (4.7)	23.2 (4.8)	22.6 (4.5)	21.9 (4.5)	.00
	Control	25.0 (4.9)	24.0 (3.9)	23.4 (4.6)	22.9 (3.5)	22.4 (3.2)	21.5 (3.2)	.00
	*p* ^c^	.57	.43	.35	.59	.68	.48	.57^b^

Results are means ± SDs. Total number of patients present in each group at each time point without intubation

*NIV* noninvasive ventilation, *PaO*_*2*_ partial pressure of arterial oxygen, *FiO*_*2*_ fraction of inspired oxygen

^a^*p* for overall comparisons of differences in each group over time

^b^*p* for overall comparisons of differences between groups over time

^c^*p* for comparisons of differences between groups at each time point

### Comparison of failure and successful cases in the NIV group

**Fig. 5:**
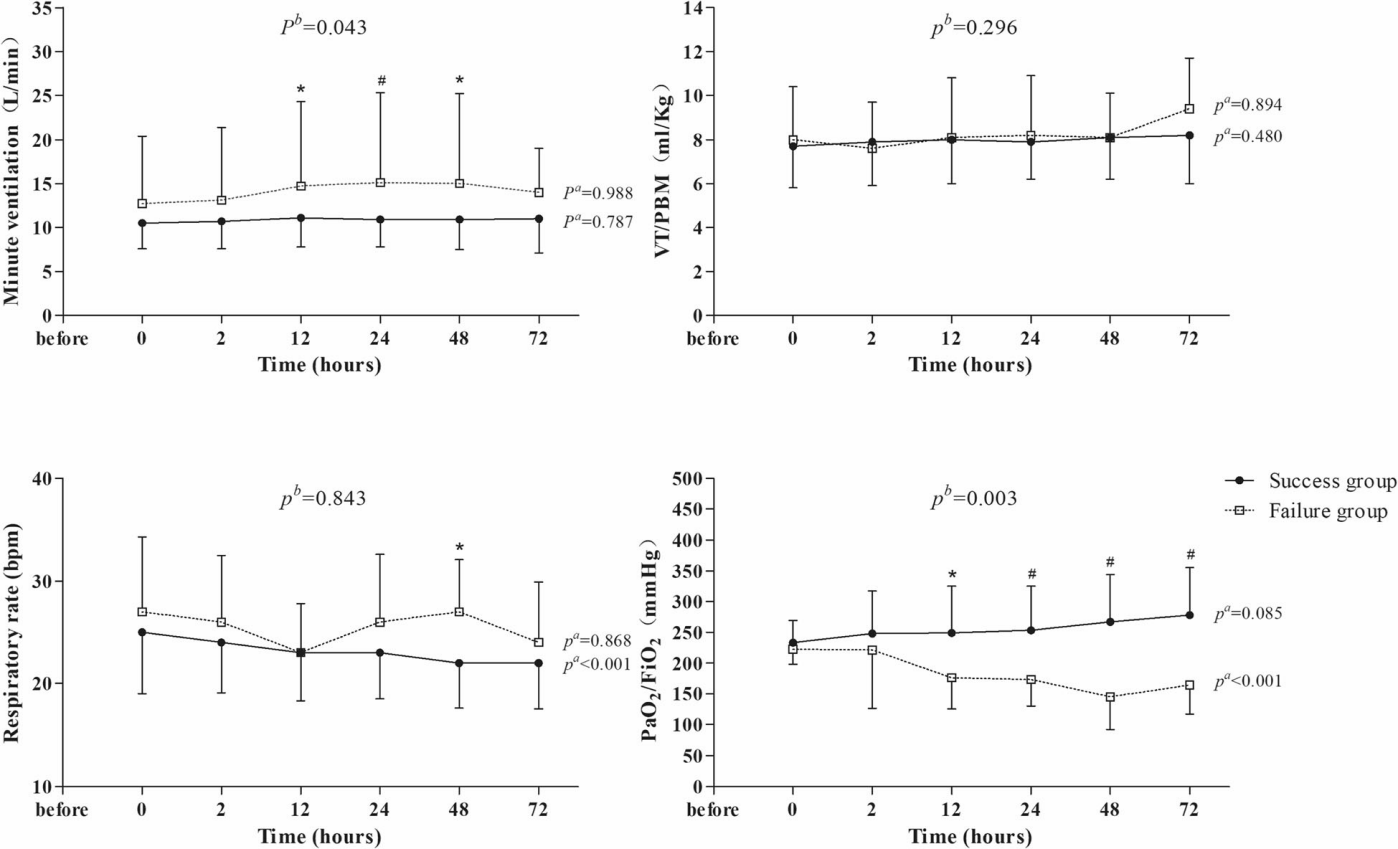
Comparisons of physiological parameters between noninvasive ventilation success and failure groups

As shown in Table 4 and Fig. 5, PaO_2_/FIO_2_ was significantly lower in failure patients than in success patients in the NIV group at 12 h after randomization and remained lower for the first 72 h. The respiratory rate was significantly higher in failure patients than in success patients at 48 h after randomization. Tidal volume per ideal body weight was higher in failure patients, but did not show significant differences. Minute ventilation (MV) showed a trend toward greater increase in failure patients than in success patients from the randomization and reached significant differences after 12 h. Univariate analysis showed albumin less than 30 g/L and RR > 25 breaths per minute at admission and MV more than 11 L/min at 12 h, 24 h, and 48 h were risk factors for NIV failure. Multivariate analysis demonstrated that MV more than 11 L/min at 48 h was the sole independent risk factor for NIV failure (OR, 1.176; 95% CI, 1.005–1.379). During the first 2 to 12 h, PaO2/FiO2 ratio did not improve, and with a high MV trend for patients in the NIV failure group compared to the successes group (Tables 5 and 6).

**Table 4:** Comparisons of physiological parameters between noninvasive ventilation success and failure groups

Variables	Group	Baseline	2 h	12 h	24 h	48 h	72 h	*p* ^a^
PaO_2_/FiO_2_, mmHg	Failure	222 (24)	221 (95)	176 (51)	173 (43)	145 (53)	164 (47)	.09
	Success	233 (36)	248 (69)	249 (76)	253 (72)	267 (77)	278 (77)	.00
	*p* ^c^	.34	.25	.02	.005	.001	.004	.003^b^
pH	Failure	7.42 (0.08)	7.41 (0.09)	7.46 (0.03)	7.45 (0.04)	7.45 (0.05)	7.45 (0.09)	.71
	Success	7.45 (0.05)	7.44 (0.04)	7.44 (0.04)	7.36 (0.79)	7.44 (0.05)	7.44 (0.04)	.46
	*p* ^c^	.28	.32	.21	.77	.63	.80	.73 ^b^
PaCO_2_, mmHg	Failure	35 (6.3)	34 (10.6)	35 (7.0)	41 (12.7)	42 (16.1)	41 (9.6)	.59
	Success	34 (5.7)	35 (5.2)	37 (10.4)	35 (5.8)	36 (5.0)	37 (5.1)	.07
	*p* ^c^	.68	.80	.55	.26	.47	.46	.23^b^
VT/PBW, mL/kg	Failure	8.0 (2.4)	7.6 (2.1)	8.1 (2.7)	8.2 (2.7)	8.1 (2.0)	9.4 (2.3)	.89
	Success	7.7 (1.9)	7.9 (2.0)	8.0 (2.0)	7.9 (1.7)	8.1 (1.9)	8.2 (2.2)	.48
	*p* ^c^	.67	.60	.94	.73	.99	.28	.30 ^b^
RR, breaths/min	Failure	27 (7.3)	26 (6.5)	23 (4.8)	26 (6.6)	27 (5.1)	24 (5.9)	.89
	Success	25 (6.0)	24 (4.9)	23 (4.7)	23 (4.5)	22 (4.4)	22 (4.5)	< .001
	*p* ^c^	.49	.22	.79	.12	.03	.35	.84^b^
Minute ventilation, L/min	Failure	12.7 (7.7)	13.1 (8.3)	14.7 (9.6)	15.1 (10.2)	15.0 (10.2)	14.0 (5.0)	.99
	Success	10.5 (2.9)	10.7 (3.1)	11.1 (3.3)	10.9 (3.1)	10.9 (3.4)	11.0 (3.9)	.79
	*p* ^c^	.06	.07	.02	.01	.02	.14	.04^b^

Results are means ± SDs

*NIV* noninvasive ventilation, *VT/PBW* tidal volume/predicted body weight, *RR* respiratory rate, *PaO*_*2*_ partial pressure of arterial oxygen, *FiO*_*2*_ fraction of inspired oxygen

^a^*p* for overall comparisons of differences in each group over time

^b^*p* for overall comparisons of differences between groups over time

^c^*p* for comparisons of differences between groups at each time point

**Table 5:** Risk factors associated with NIV failure in univariate analysis

Variable	Wald	Odds ratios	95% confidence interval	*p*
RR > 25 bpm at 48 h	5.767	15.873	1.661–142.857	0.02
MV > 11 L/min at 12 h	4.163	1.156	1.006–1.330	0.04
MV > 11 L/min at 24 h	5.060	1.170	1.020–1.340	0.02
MV > 11 L/min at 48 h	4.079	1.176	1.005–1.379	0.04
albumin < 30 g/L at admission	7.397	18.437	2.257–150.591	0.01

*NIV* noninvasive ventilation, *MV* minute ventilation

**Table 6:** Risk factors associated with NIV failure in multivariate analysis

Variable	Wald	Odds ratios	95% confidence interval	*p*
MV > 11 L/min at 48 h	4.079	1.176	1.005–1.379	0.043

*NIV* noninvasive ventilation, *MV* minute ventilation

## Discussion

To our knowledge, the present study is the first and largest randomized controlled trial to evaluate NIV for patients with early pneumonia-induced mild ARDS. The main strength of our study is its high homogeneity with only pneumonia-induced mild ARDS patients included. The major finding of our study revealed that, compared to the Venturi mask, NIV did not reduce the need for intubation or mortality in pneumonia-induced early mild ARDS.

The rate of the need for intubation is lower than expected in our study. This may reflect patients being included in a very early stage of mild ARDS. In a previous study, timing for NIV application was based on a simple three-component early acute lung injury score (1 point for oxygen requirement > 2–6 L/min or 2 points for > 6 L/min; 1 point each for a respiratory rate ≥ 30 and immune suppression [EALI score]). A score greater than or equal to 2 points identified patients who progressed to ALI and requiring NIV [16].The average respiratory rate in our early mild ARDS patients was 25, which suggests that our patients may had a less severe ARDS than that in the EALI study. RR used as selection criteria is helpful for including patients with more severity, especially with our finding that high minute ventilation was associated with NIV failure. Unfortunately, RR was not used as an inclusion criterion in our study which may be a reason for a low intubation rate and mortality.

Different pneumonia pathogens may be another reason for a relatively low intubation rate in our study. Bacterial pneumonia has a high possibility to progress to sepsis and related severe lung injury [17]. Another study [2] of pneumonia-induced hypoxemic acute respiratory failure (hARF) patients supported with CPAP included both CAP and HAP, a positive culture in about 50%, indicating bacterial infection. However, in our study, most cases were CAP, and only 10–15% were culture positive, suggesting a lower proportion of bacterial pneumonia-induced respiratory failure in our patients. Therefore, the type of pneumonia and the likelihood of bacterial etiology may result in different rates of progression to more severe ARDS and more need for intubation.

The primary outcome analysis of our study showed no difference in the need for intubation between the NIV and control groups. This may reflect the lack of recruitment responsiveness to NIV positive airway pressure in early mild ARDS patients. A meta-analysis revealed that higher airway pressure levels were associated with improved survival among the subgroup of ARDS patients with PaO_2_/FIO_2_ less than 200 mmHg [18], who demonstrate better recruitment with positive airway pressure. In our study, we included patients with a PaO_2_/FIO_2_ higher than 200 mmHg, who may be less responsive to NIV, leading to a negative result for NIV compared to conventional oxygen therapy. PaO_2_/FIO_2_ was significantly higher in the NIV group than in the control group at 2 h after inclusion, and this trend remained for the first 72 h, similar to previous studies [13, 19]. However, despite an initial improvement of arterial hypoxemia, the use of NIV did not result in changes of the intubation rate nor outcome variables in our study. In a recent large trial of immunocompromised patients admitted to the ICU with hARF, early NIV also did not reduce the incidence of intubation or mortality compared with oxygen therapy alone [20]. However, the median duration of NIV in this study was 8 h within the first 24 h, 6 h on day 2, and 5 h on day 3. This negative study therefore may represent insufficient support time for NIV therapy. In our study, average NIV duration was more than 16 h per day. Despite this support time dose, we did not show a positive effect on avoidance of intubation. Finally, the management of continue NIV in the NIV group or crossover to NIV in the control group after the patients met the intubation criteria may influence the final outcome such as mortality, length of ICU or hospital stay, or complications.

Such a long period of NIV support through a facial mask may affect the patient comfort. A recent study showing that an NIV helmet could reduce intubation in patients with ARDS [21]. The comfort of patients with face mask was evaluated in our study with a previously reported method [2, 22, 23], and only one patient ceased the NIV because of intolerance.

Our results indicate that a minute ventilation exceeding 11 L/min may be a predictor of NIV failure. A recent clinical trial suggests that NIV administered to patients with severe lung injury could increase ventilator-induced lung injury by generating tidal volumes that exceeded 9 mL per kilogram predicted body weight [24, 25]. However, the VT/PBW was between 7.7 and 9.4 mL/kg in the present study. A low expired tidal volume is almost impossible to achieve in the majority of patients receiving NIV for acute hypoxemic respiratory failure. The high tidal volume resulting from the high respiratory drive in these patients may lead to lung injury and NIV failure [26]. High tidal volume and minute ventilation were also found in NIV patients in LUNG SAFE study [10]. And in FLORALI study, NIV did not result in significantly different intubation rates compared to standard oxygen in patients with non-hypercapnic hARF, and the intubation rate in NIV was even higher than standard oxygen. This study suggests that high flow humidified nasal cannula (HFNC) may be more beneficial than NIV or standard oxygen [24]. This may also be explained by lung injury caused by high driving pressure during NIV. Based on our data, the parameter of MV should be monitored for a limitation of less than 11 L/min in early mild ARDS.

The differences in PaO_2_/FIO_2_ and minute ventilation between NIV failure and success patients, shown after 12 to 48 h of NIV application, are similar to a failure time of 1 to 48 h after NIV initiation reported by Ozyilmaz et al. [27]. In the NIV group of our study, mean delay between inclusion and failure was almost 5 days, and longer than 2.6 days in the control group. At the intubation time point, the NIV group has a worse state than the control group with lower PaO_2_/FiO_2_ (120 mmHg vs. 147 mmHg) and higher RR, which may suggest a delay in intubation by use of NIV. Furthermore, we noticed that, during the first 2 to 12 h after inclusion, PaO_2_/FiO_2_ ratio did not improve, and with a high MV trend for patients in the NIV failure group compared to the success group. This may be an early predictor for NIV failure for the pneumonia-induced mild ARDS. And recently, a HACOR score was proposed for patients with NIV failure in hypoxic patients [28]. HACOR score improves in patients with NIV success and remains unaltered in patients with NIV failure, which also emphasized the trend of the five predictors from the score is important for predicting NIV failure.

Severe pneumonia is frequently of acute onset, demonstrates bilateral infiltrates on chest radiography, and causes severe acute respiratory failure not due to cardiac failure. Thus, differentiating severe bilateral pneumonia from ARDS is virtually impossible on clinical grounds alone. The differentiation of severe bilateral pneumonia from pneumonia-induced ARDS may be based on the measurement of decreased compliance in invasive ventilation, on a lung biopsy finding of diffuse alveolar damage (DAD), a complicated septic shock with pneumonia, or evidence of viral etiology. However, these criteria cannot be applied to the mild non-intubated ARDS patients included in our study. Therefore, we cannot exclude the possibility that specific sub-phenotypes of pneumonia patients are more or less responsive to NIV.

The last possibility for our findings is that early in pneumonia-induced mild ARDS, appropriate and effective anti-infection therapy may be more important than oxygenation and ventilation strategies. Greater culture positivity, and therefore presumed correctly adjusted antibiotics, is associated with improved outcomes with NIV for pneumonia. Others have demonstrated the early appropriate antibiotics are associated with less progression to ARDS in patients admitted with pneumonia [29].

The main limitation of our study was that the definition of early mild ARDS was based on the American-European consensus conference criteria for ALI. Patients did not receive positive pressure at inclusion assessment. This results in our patients having lower severity of mild ARDS than those meeting the Berlin definition. Inclusion of pneumonia patients with very early stage of mild ARDS may have resulted in lower progression to ARDS and the need for intubation than expected. Although sputum culture was routinely performed for every patient, the positive culture rate is low. However, most patients were treated with guideline-compliant antibiotics and improved. The recruitment rate was also slower than expected because of a strict enrollment and exclusion criteria, potentially leading to time bias over the course of the study. Finally, based on the low incidence of intubation in our study, the sample size may be under power, and a sample size reevaluated as about 3000 cases in total may be needed for a settled conclusion.

## Conclusions

In conclusion, treatment with NIV did not reduce the need for intubation among patients with pneumonia-induced early mild ARDS, despite the improved PaO_2_/FIO_2_ observed with NIV compared with standard oxygen therapy. High minute ventilation may predict NIV failure.

## Additional file

Additional file 1: Online Methods Supplement; Tables; **Figures 1.** Online Methods Supplement. 1.1 Section S1: Study Subjects: Inclusion and Exclusion Criteria. 1.2 Section S2: Randomization. 1.3 Section S3: Blinding and Quality Control. 1.4 Section S4: Study Methods. 2. Online Tables: 2.1 Table S1: Subgroup Analysis. 1.2 Table S2: Inclusion Number for Each Center per Year. 3. Online Figures: 3.1 Figure S1: The Daily Ventilation Time of NIV. 3.2 Figure S2: The maximum levels of IPAP (above EPAP) and EPAP Use during NIV. (PDF 376 kb)

## Data Availability

The datasets generated and analyzed during the current study are publicly available at the website of our database and are also available from the corresponding author on reasonable request.

## Abbreviations

ALI: Acute lung injury
ARDS: Acute respiratory distress syndrome
NIV: Noninvasive ventilation
MV: Minute ventilation
