# Supplementary material for: A multicenter RCT of noninvasive ventilation in pneumonia-induced early mild acute respiratory distress syndrome
Source: Crit Care. 2019 Sep 4;23:300. doi: 10.1186/s13054-019-2575-6 (PMC6727327; doi:10.1186/s13054-019-2575-6)
Supplement: Supplementary file 1 — Online Methods Supplement; Tables; Figures 1. Online Methods Supplement. 1.1 Section S1: Study Subjects: Inclusion and Exclusion Criteria. 1.2 Section S2: Randomization. 1.3 Section S3: Blinding and Quality Control. 1.4 Section S4: Study Methods. 2. Online Tables: 2.1 Table S1: Subgroup Analysis. 1.2 Table S2: Inclusion Number for Each Center per Year. 3. Online Figures: 3.1 Figure S1: The Daily Ventilation Time of NIV. 3.2 Figure S2: The maximum levels of IPAP (above EPAP) and EPAP Use during NIV. (PDF 376 kb) [file 13054_2019_2575_MOESM1_ESM.pdf]

Additional file 1: Online Methods Supplement; Tables; Figures

1. Online Methods Supplement
1.1 Section S1: Study Subjects: Inclusion and Exclusion Criteria
1.2 Section S2: Randomization
1.3 Section S3: Blinding and Quality Control
1.4 Section S4: Study Methods

2. Online Tables:
2.1 Table S1: Subgroup Analysis
1.2 Table S2: Inclusion Number for Each Center per Year

3. Online Figures:
3.1 Figure S1: The Daily Ventilation Time of NIV
3.2 Figure S2: The maximum levels of IPAP (above EPAP) and EPAP Use during NIV

## 1. Online Methods Supplement

### 1.1 Section S1: Study Subjects: Inclusion and Exclusion Criteria

**The diagnostic criteria for pneumonia** were infiltrates or interstitial change in chest radiograph, with or without pleural effusion and at least one of the following criteria were met: 1) presentation of cough, sputum or exacerbation of previous respiratory symptoms, with or without chest pain; 2) Fever; 3) white cell count more than 10×10^9/L or less than 4×10^9/L. And tuberculosis, tumor, non-infectious interstitial lung diseases, cardiac pulmonary edema, atelectasis, pulmonary embolism, eosinophilic lung disease and vasculitis should be excluded.

All patients admitted with pneumonia (*see* criteria in the online supplement) were screened and patients who selected by the inclusion and exclusion criteria were included.

Study physicians screened all adult chest radio graphs or chest CT when they admitted to Hospital. When a qualifying chest radiograph or CT was identified, the primary author (H.H) reviewed all films prior to enrollment (as well as all films qualifying for early mild ARDS). Patients admitted with an abnormal chest radiograph not meeting criteria (i.e., unilateral abnormalities or a reading of minimal bibasilar opacities without other signs or symptoms of lung injury) were followed and enrolled if they progressed to a subsequent qualifying film within 72 hours.

**The diagnostic criteria for early mild ARDS and inclusion criteria** were: 1) pneumonia; 2) acute onset, less than 7 days; 3) 200 mm Hg < $PaO_2/FIO_2$ < 300 mm Hg while breathing oxygen delivered by a conventional Venturi device at a fraction of inspiration oxygen of 0.5; 4) presence of bilateral pulmonary infiltrates on posteroanterior chest radiograph or chest computed tomography (CT); and 5) no evidence of left atrial high pressure as assessed by echocardiography shown left atrial enlargement and/or no evidence of left heart failure as assessed by a pulmonary artery wedge pressure of >18 mm Hg (9).

**The exclusion criteria** were: 1) age <18 yrs; 2) PaCO2 >50 mmHg; 3) Glasgow Coma Scale <11; 4) airway or facial injury; 5)pneumothorax or pneumomediastinum; 6) unable to spontaneously clear secretions from the airways; 7) respiratory arrest; 8) severe ventricular arrhythmia or unstable myocardial ischemia; 9) severe organ dysfunction (Sequential Organ Failure Assessment score >=3) ; 10) end-stage patients who were expected to survive < 6 months; 11) severe abdominal distension; 12) refusal to receive NIV; 13) unable to cooperate with NIV application; 14) chronic obstructive pulmonary disease; 15) interstitial lung diseases; and 16) ARDS caused by extra-pulmonary reasons, such as extra-pulmonary infection related sepsis, severe acute pancreatitis, severe multiple trauma, drug overdose, and blood transfusion.

### 1.2 Section S2: Randomization

**Randomization**: A centralized interactive contact system was used for randomization. The random block length was 4, and random numbers were generated by computer. All of the centers participating in this study were immediately put in contact with the central unit (Beijing Chao-Yang Hospital) to obtain a randomization number if a patient fulfilled the inclusion criteria. Within 24 hrs of fulfilling inclusion criteria, a patient was randomly allocated either to the NIV group or the control group (Venturi mask oxygen therapy).

## 1.3 Section S3: Blinding and Quality Control

The trial was overseen by a steering committee, and data quality control was completed by independent data monitoring board. Clinicians and epidemiologists of above organization were not members in our research group. Research assistants timely verified online database and regularly monitored all the centers on site to ensure the accuracy of the data recorded. An investigator at each center was responsible for enrolling patients in the study, ensuring adherence to the protocol, and completing the electronic case-report form. Although the individual study assignments of the patients could not be masked, the coordinating center and all the investigators remained unaware of the study group outcomes until the data were locked in June 2015. All the analyses were performed by the study statistician who did not participate in the study and did not know the research grouping.

## 1.4 Section S4: Study Design and Methods

**Noninvasive ventilation management**:

Patients in the NIV group were ventilated using the bilevel positive airways pressure S/T mode (BiPAP Vision or V60; Respironics Inc., Murrysville, PA). A face mask (ZS-MZ-A Face Mask; Shanghai Zhongshan Medical Technology Co., Shanhai, China) was used for all patients.

Expiratory positive airway pressure(EPAP) was initially set at 4 cm $H_2O$ and increased by 1–2-cm $H_2O$ increments up to a patient's maximum tolerance.

$FIO_2$ was set to maintain $SpO_2$ at 92% to 96%. If the $SpO_2$ was < 92% at 10–12 cm $H_2O$ or a patient's maximum tolerance for the EPAP level, $FIO_2$ could be increased to > 0.6.

Inspiratory positive airway pressure(IPAP) was adjusted by increments of 2 cm H2O every 5 to 6 mins to obtain a tidal volume between6 mL/kg and 10 mL/kg for each patient.

NIV was continuously delivered after entry into the study for no less than 16 hours a day in the first 3 days. Disconnecting from the ventilator for short periods was allowed to clear secretions, to drink water, or to eat food but was not scheduled. Patient tolerance to NIV, was assessed by means of a scale used and validated in previous studies (CCM,2000.PMID: 10890620) that is defined as follows: 1, bad; 2, poor; 3, sufficient; 4, good; 5,very good. The patients were asked by the respiratory therapist to answer the following question: "How do you feel with your breathing?" The patient gave the score to the therapist at the end of each run.

When patients received $FiO_2$ < 0.40, attempts to withdraw NIV were made if they achieved $SpO_2$ of > 92% for 24 hrs while spontaneously breathing Venturi oxygen at $FIO_2$ = 0.35.

**The modified scale of accessory respiratory muscle use** were

1 = no visible sternocleidomastoid activity

2 = sternocleidomastoid activity without active contraction of the supraclavicular or intercostal muscles

3 = vigorous activity of accessory muscles with contraction

4 = vigorous activity with contraction of accessory muscles and a paradoxic abdominal breathing pattern.

**The anti-infection protocols.**

1. Community-acquired pneumonia: β-lactams combined with macrolides or fluoroquinolones alone.

2. Hospital-acquired pneumonia or ventilator-associated pneumonia: β-lactams which have anti-Pseudomonas aeruginosa activity and can be combined with the respiratory quinolones or aminoglycosides. If high-risk factors or evidence of methicillin-resistant Staphylococcus aureus infection are present, then glycopeptides or linezolid can be combined.

3. Opportunistic infections in patients with immunodeficiency: If risk factors or evidence of cytomegalovirus infection are present, then ganciclovir can be used. If risk factors or evidence of human Pneumocystis jirovecii infection exist, then sulfonamide antibiotics can be used. If evidence of invasive fungal infection are present, sensitive azoles, amphotericin B or echinocandins can be used.

4. Influenza: During the influenza epidemic season, sensitive antiviral drugs (i.e., oseltamivir) can be used when evidence of influenza virus infection is detected.

**Comprehensive therapy**

Comprehensive therapy was provided by the ICU attending physicians based on published guidelines and protocols including enhancing body immunity, protect the functions of gastrointestinal tract, prevent thromboembolism, maintaining water and electrolyte acid-base balance, glycemic control, and nutrition support.

**Data and sample collection**

The characteristics and blood samples and sputum samples for all patients were collected and tested for the diagnosis and pathogenesis of pneumonia and whole blood cell count, ABG, biochemical and coagulation indexes were done at the inclusion. Endotracheal aspiration or BALF samples for bacterial and fungal culture were collected in intubated patients.

## 2. Tables

2.1 Table S1: Subgroup Analysis for white blood cell count, neutrophil cell percentage, respiratory rate, state of immunocompromised, procalcitonin level, and types of pneumonia at inclusion.

| | NIV group | control group | *P* value | NIV group | control group | *P* value |
|---|---|---|---|---|---|---|
| WBC at inclusion | WBC<10×10$^9$ | | | WBC>10×10$^9$ | | |
| | n=51 | n=57 | | n=51 | n=41 | |
| Number of patients need for intubation,no(%) | 5(9.8) | 5（8.8） | 1.000 | 6(11.8) | 4（9.8） | 1.000 |
| N%<70% at inclusion | N<70% | | | N>70% | | |
| | n=12 | n=10 | | n=90 | n=88 | |
| Number of patients need for intubation,no(%) | 1(8.3) | 0(0.0) | 1.000 | 10(11.1) | 9(10.2) | 0.850 |
| RR at inclusion | RR<25 bpm | | | RR>25 bpm | | |
| | n=63 | n=62 | | n=39 | n=36 | |
| Number of patients need for intubation,no(%) | 5(7.9) | 6（9.7） | 0.731 | 7(17.9) | 3（8.3） | 0.313 |
| Immunocompromised at inclusion | Immunocompromised | | | Immunocompetent | | |
| | n=9 | n=10 | | n=93 | n=88 | |
| Number of patients need for intubation,no(%) | 2(22.2) | 0（0.0） | 0.211 | 9(9.7) | 9（10.2） | 0.902 |
| PCT>0.5 at inclusion | PCT>0.5 | | | PCT<0.5 | | |
| | n=59 | n=53 | | n=43 | n=45 | |
| Number of patients need for intubation,no(%) | 7(11.9) | 4(7.5) | 0.443 | 4(9.3) | 5(11.1) | 0.780 |
| Type of pneumonia at inclusion | CAP | | | HAP | | |
| | n=95 | n=93 | | n=7 | n=5 | |
| Number of patients need for intubation,no(%) | 10(10.5) | 9（9.7） | 0.847 | 1(14.3) | 0（0.0） | 1.000 |

WBC,white blood cell count; N%, neutrophil cell percentage; RR, respiratory rate; PCT, procalcitonin level; CAP, community acquired pneumonia; HAP, hospital acquired pneumonia.

2.2 Table S2: Inclusion Number for Each Center per Year

| Center | Number of patients included per year | | | | |
|---|---|---|---|---|---|
| | 2012 | 2013 | 2014 | 2015 | total No. for each center |
| Beijing Chao-Yang Hospital | 2 | 3 | 5 | 1 | 11 |
| Beijing Hospital | 2 | 1 | | | 3 |
| Affiliated Hospital of Logistics College of Chinese Armed Police Forces | 2 | 10 | 8 | 6 | 26 |
| The First Affiliated Hospital of Chongqing Medical University | 5 | 8 | 7 | | 20 |
| The First Affiliated Hospital of Wenzhou Medical University | 3 | 11 | 4 | 1 | 19 |
| Fujian Province Hospital | 1 | 6 | 6 | 3 | 16 |
| The Third Affiliated Hospital of Inner Mongolia Medical College | 6 | 7 | 1 | 2 | 16 |
| Xijing Hospital of the Forth Military Medical University | 1 | 6 | 6 | | 13 |
| The Third People’s Hospital of Chengdu | 5 | 3 | 5 | | 13 |
| Chengdu Fifth People’s Hospital | 1 | 4 | 4 | | 9 |
| People’s Hospital of Beijing Daxing District | 4 | 2 | 2 | | 8 |
| Chinese PLA General Hospital | | 5 | 2 | 1 | 8 |
| The First Affiliated Hospital of Xi’an Jiaotong University | | | 6 | 1 | 7 |
| Lung Disease Hospital of Fujian Fuzhou | | 1 | 4 | 1 | 6 |
| The Second Affiliated Hospital of Chongqing Medical University | | 1 | 5 | 1 | 7 |
| Beijing Tongren Hospital | | 1 | 4 | 1 | 6 |
| Tangdu Hospital, the Fourth Military Medical University | 2 | 2 | | | 4 |
| Peking University Third Hospital | 1 | | 3 | | 4 |
| Xinqiao Hospital Army Medical University | 2 | | 1 | | 3 |
| People's Hospital of Xinjiang Uygur Autonomous Region | 1 | 1 | 1 | | 3 |
| General Hospital of Ningxia Medical University | | 1 | | 1 | 2 |
| Total No for each year | 38 | 73 | 74 | 19 | 204 |

## 3. Figures

**3.1 Figure S1 The Daily Ventilation Time of NIV.**

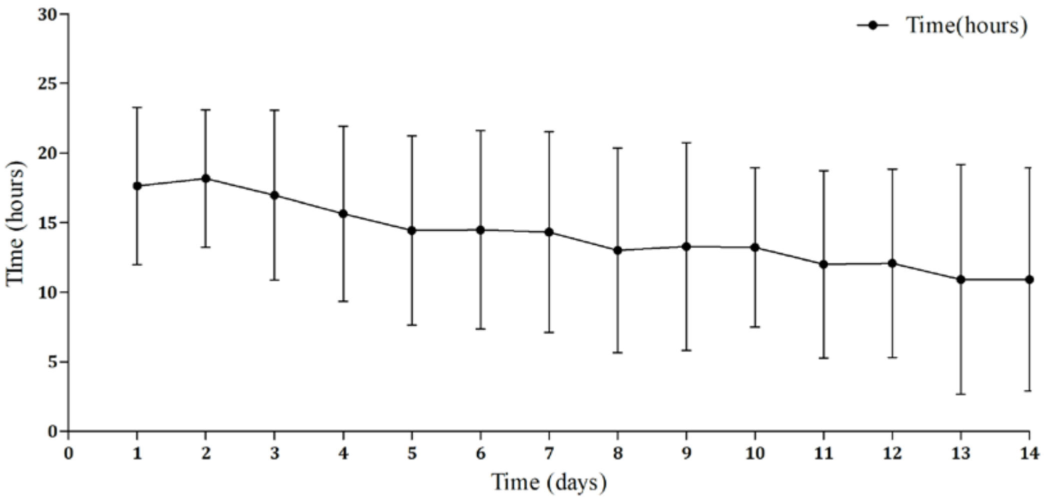

**3.2 Figure S2: The maximum levels of IPAP and EPAP Use during NIV.**

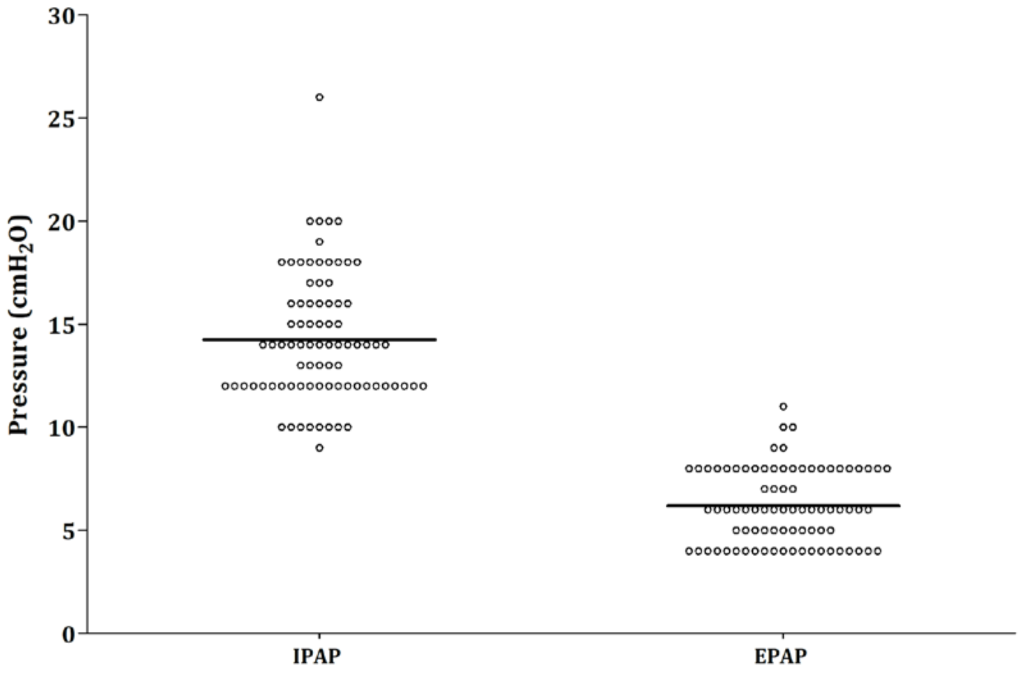


Legends for Figure S2: The maximum levels of IPAP and EPAP Use during NIV.

The maximum IPAP level and EPAP level for each patient were shown in circle points, and the average pressure levels were shown as in lines.
